# Resolving agrochemical penetration in wheat leaves with secondary ion mass spectrometry imaging and depth profiling

**DOI:** 10.1007/s00216-025-06134-1

**Published:** 2025-09-30

**Authors:** Akhila Ajith, Sadia Sheraz, Aline Xavier de Souza, Drupad K. Trivedi, Jean-Yves Mugnier, Giles N. Johnson, Phillip J. Milnes, Nicholas P. Lockyer

**Affiliations:** 1 Photon Science Institute, Department of Chemistry, University of Manchester, Manchester, UK; 2 Syngenta, Jealott’s Hill International Research Centre, Bracknell, UK; 3 Manchester Institute of Biotechnology, Department of Chemistry, University of Manchester, Manchester, UK; 4 Department of Earth and Environmental Sciences, University of Manchester, Manchester, UK

**Keywords:** Secondary ion mass spectrometry, Agrochemicals, Depth distribution

## Abstract

The foliar application of an agrochemical is first encountered by the hydrophobic cuticle and then the hydrophilic epidermis of the leaf. Understanding the movement and distribution of pesticides through these layers is vital in the research and development process of agrochemicals. Currently, no technique provides conclusive answers on active ingredient penetration through the leaf surface layers without chemical or physical treatment of the leaves. The advancements in modern time-of-flight secondary ion mass spectrometry (ToF-SIMS) technologies provide the necessary depth, spatial, and mass resolution to probe this complex area of interest with precision and relatively simple sample preparation. In this work, we describe a systematic ToF-SIMS workflow to comprehensively understand the foliar movement of an applied agrochemical through the surface waxes and in the bulk of the leaf. A combination of depth profiling experiments along with cross-section imaging of agrochemical-applied wheat leaves was used to better understand the mobility of a fungicide, azoxystrobin, in young wheat leaves. Extensive testing was done to optimise the analytical conditions and sample preparation strategies best suited for the experiments described in this work. A gas cluster ion beam composed of water clusters was selected for analysis. Freeze-dried leaves were used for depth profile experiments, while tape-sectioned leaf cross-sections were used for imaging. Parallel leaf sections to those taken for ToF-SIMS were also imaged with matrix-assisted laser desorption ionisation mass spectrometry imaging (MALDI-MSI). When applied, azoxystrobin moved rapidly across the leaf cross-section, being observed in the leaves as early as 24 h after application and persisting for 1 week. When comparing the intensity in the mesophyll region to that of the vascular bundle, more azoxystrobin content was observed in the mesophyll at both time points, with greater disparity observed at 1 week. The results obtained were corroborated with the MALDI study done on similar samples and with pre-existing literature.

**Graphical Abstract:**

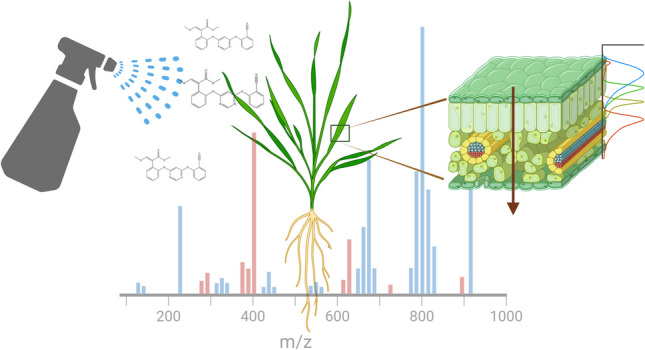

**Supplementary Information:**

The online version contains supplementary material available at 10.1007/s00216-025-06134-1.

## Introduction

Achieving global food security, while adapting to the constraints of a changing climate and land use patterns, is a major challenge in our society [1]. The use of agrochemicals can have a major impact on food production, providing better crop yields per available land area by reducing the effects of pests, microbes, and other biotic/abiotic stresses affecting plants. Pesticide usage helps control crop losses, protecting approximately 78% of fruits, 54% of vegetables, and 32% of cereals [2]. However, there have been increasing reports on the potential toxicological and environmental impact of the use of crop protection products [3]. Thus, it is important to have strict agrochemical testing in place along with the use of advanced technologies to gain comprehensive insights into the behaviour and kinetics of applied compounds within plants.

The cuticle is the lipophilic barrier of outer primary plant surfaces when faced with environmental stress and pathogen attacks. This protective shield is a key evolutionary step to plants’ terrestrialisation, limiting water loss to the atmosphere and providing a defence against environmental stresses, such as herbivore and pathogen attacks [4, 5]. A main source of carbon for fungal species is living or dead plant tissue, and there are different interactions of the fungal species with plants that enable it, including symbiosis, saprotrophy, and parasitism. Fungal pathogens infecting leaves must penetrate the leaf cuticle to gain access to the nutritional content of the inner cells [6]. Hence, the leaf cuticle and epidermal cells are a major hub for plant-fungal interactions and comprise a crucial region for the study of foliar fungicide application and preventative treatments against pathogenic fungi. A similar scenario of surface interactions exists for all foliar-applied pesticides and the pests/pathogens involved. However, no mainstream techniques currently used in the agrochemical industry provide the required spatial and depth resolution or the chemical specificity for such studies without chemical or physical treatment of the samples.

Secondary ion mass spectrometry (SIMS) is one of the most versatile mass spectrometry techniques currently, with the capability to produce chemical 2-D images, 3-D models, and depth profiles of a variety of samples [7]. The advent of polyatomic primary ion beams [8, 9] has enabled in-depth analysis and higher sensitivity over extended mass ranges, widening the capabilities of SIMS from sub-micron analysis of nanoscale materials [10] to single-cell spatial omics applications [11].

The efficacy of an applied agrochemical will depend on its mobility through the wax layers into the plant tissue and the time scales of this movement. Traditionally, the wax retention and tissue penetration of pesticides have been elucidated by independently analysing chemically stripped wax and macerated leaf bulk with techniques including LC-MS [12]. There have been some mass spectrometry imaging (MSI) studies investigating agrochemical distribution in plant leaves with desorption electrospray ionisation (DESI) MSI or matrix-assisted laser desorption ionisation (MALDI) MSI [13, 14]. MSI methodology does not require radio-labelled analogues of the applied agrochemical for spatial mapping, unlike autoradiography, the current gold standard in the agrochemical industry for spatial localisation studies and can image multiple chemicals in one single experiment. Yet, due to the technical and/or sample preparation limitations of DESI and MALDI-MSI techniques, they have been restricted to either surface distribution studies [15] or relatively low-resolution cross-section imaging of sub-millimetre-thick leaves [16]. The ToF-SIMS technique, with its molecular range, sensitivity, and spatial and depth resolution, provides unique data to better understand this region of the plant leaves. The depth profile data, along with the cross-section images obtained with ToF-SIMS, can help provide a better understanding of how an active ingredient navigates these chemically distinct layers and dissipates into the underlying leaf tissue. However, to the best of our knowledge, no studies have explored the possibilities of depth profiling combined with an imaging modality to visualise the agrochemical penetration in plant leaves with ToF-SIMS.

In this work, we have done a comprehensive study to optimise ToF-SIMS analytical conditions, including primary ion beam conditions and sample preparation strategies for understanding the fungicide penetration in wheat leaves through depth profiling and imaging. Wheat was selected for this study as it is the most cultivated target crop of azoxystrobin, and a widely used systemic fungicide formulation containing azoxystrobin was selected for this study [17]. When applied to the foliage, azoxystrobin penetrates through the leaf cuticle to enter the plant tissue. Once inside, it is distributed throughout the plant, inhibiting fungal respiration and providing protection against fungal pathogens from within the plant tissues [18].

## Materials and methods

### Chemicals and reagents

Syngenta Ltd. provided the azoxystrobin formulation, AMISTAR SC (AZ formulation) and the azoxystrobin standard was obtained from Sigma-Aldrich, UK. All other chemicals and UHPLC or HPLC grade solvents were acquired from commercial sources unless stated otherwise.

### Sample preparation of azoxystrobin formulation

To determine the optimum water cluster ion beam for this study, 10 µL of AZ formulation (2500 ppm) in water was drop-cast onto silicon wafers and allowed to air dry. To understand the limit of detection of the azoxystrobin formulation, 10 µL of different concentrations of the AZ formulation in water (250 ppm, 25 ppm, 2.5 ppm, 0.25 ppm, blank) was used. Two samples were prepared for each concentration. The silicon wafers were then stuck to the SIMS sample holder using a double-sided carbon tape and taken for analysis.

### Wheat plants

The study used young wheat plants 10 to 15 days after planting the seeds, typically in the two-leaf stage. Tybalt variety of wheat seeds (Limagrain, Lincolnshire, UK) were planted on Levington’s multi-purpose compost. The temperature conditions of 18/22 °C for night/day were maintained with a light intensity of 100 μmol m^−2^ s^−1^ over the day length of 16 h during the growing and treatment of the plants.

### Sample preparation of wheat leaves

The wheat leaves used for testing various primary ion beams were untreated. The untreated plants were uprooted and transported to the analysis lab, maintaining pristine conditions until the experiment. A mid-portion of a wheat leaf was fixed to the sample holder with a double-sided carbon tape and was treated with 2500 ppm AZ formulation and then air-dried at room temperature for 2 h. Under SIMS analysis, whole leaves can cause charge build-up; hence, for all depth profiling studies described herein, a steel grid was placed on top of the sample to help minimise sample charging with appropriate sample bias voltage applied.

For the systematic study on the active agrochemical penetration of AZ formulation, an *in-plant* formulation application was performed. A middle area of one of the two leaves in the plant was selected for the application of the AZ formulation. At the time points of sampling, which were 24 h and 1 week after formulation application, the plants were uprooted and carefully transported to the analysis lab. The samples of the active penetration studies were analysed using ToF-SIMS depth profiling and mass spectrometry imaging.

For the depth profiling studies, the treated leaf was excised from the plant and fixed to the SIMS sample holder just before sample loading into the prep chamber of the instrument. The treated area was cut out with a sterile blade and promptly mounted on a sample holder as soon as possible to maintain the innate localisation of the applied formulation. However, the imaging studies with ToF-SIMS and MALDI require cryo-sectioning of the leaf, as we probe the whole cross-section of the leaf.

For the imaging studies, instead of fixing the sample to the stub, the excised area of interest of the leaf after formulation application was flash-frozen on a steel plate on top of dry-ice hexane slurry at around −100° C. The frozen leaf sample was embedded in a 2% v/v solution of carboxymethyl cellulose (CMC) in water using a disposable standard tissue-tek cryomold (Agar Scientific, UK). The cryomold was first partially filled with the CMC solution and submerged in dry-ice hexane slurry until the edges of the solution started to freeze. Before the solution was completely frozen, the frozen leaf was carefully fixed in the middle of the partially filled mould, filling in the rest of the cryomold with the CMC solution. After the sample block was completely frozen, it was stored in a −80° C freezer until taken for cryo-sectioning. A specialised tape-sectioning method was adapted from Kawamoto et al. [19] to maintain the chemical and structural localisation of the fragile wheat leaves. The sample was left to equilibrate in the cryotome for 1 h before sectioning. The Cryofilm type IMS(II) conductive tape (Section-Lab Co. Ltd, Yokohama, Japan) was stuck to the sample block and was sectioned with a thickness of 20 µm at −20° C sample temperature. Further details on the cryo-sectioning method optimisation can be found in Supplementary Note 2.

The cryo-sections needed to be fixed differently to appropriate substrates for ToF-SIMS and MALDI imaging due to variable constraints shown by the sample stage in both instruments. The conductive tape with the leaf section was placed on a PTFE sheet with the help of FrozenSTUCK™ cryo-compatible double-sided tape (TAG Scientific, East Sussex, UK) and was stored at −80° C until freeze-drying before SIMS analysis. For MALDI imaging, the cryo-sections on the conductive tape were stuck to a standard microscope slide with FrozenSTUCK™ tape and stored in −80° C freezer. It was observed that sample desiccation at room temperature caused visible disruption of cryo-section integrity. Therefore, the samples were freeze-dried at −50° C for 1.5 h before taking the samples for either SIMS or MALDI imaging.

### Optimising ToF-SIMS analysis conditions

The ToF-SIMS instrument (J105 3D Chemical Imager, Ionoptika Ltd, Chandlers Ford, UK) used in this study is equipped with a 40 keV C_60_^+^ primary ion beam and a 70 keV gas cluster ion beam (GCIB) composed of an Ar/CO_2_ mixture (86% Ar, 12% CO_2_, and 2% O_2_) or water. We evaluated the sensitivity of the different primary ion beams for the depth profiling of a mature wheat leaf. The leaf samples were prepared as in section “Sample preparation of wheat leaves”. The leaf was kept in the vacuum (< 10^–6^ mbar) of the instrument prep chamber to stabilise overnight. An area of 500 × 500 µm^2^ was depth profiled for 200 sputter cycles with the same ion dose of 5 × 10^11^ ions/cm^2^/sputter cycle with C_60_^+^, Ar/CO_2_ and the water cluster ion beams. For the two GCIB ion beams tested, the same energy per mass unit of 0.2 eV/u was used. The data was normalised to the total ion count per sputter cycle for comparison.

Existing literature indicates that the sensitivity of an analyte can vary with the cluster size of the GCIB or the energy per unit mass [20]. Water cluster beams with various cluster sizes (11 k to 26 k) were used on samples described in “Sample preparation of azoxystrobin formulation” to optimise the energy per unit mass (E/u) or cluster size of the beam that gives the best sensitivity for analysing azoxystrobin. A similar experimental setup was also used to understand the limit of detection of the AZ formulation. For both studies, three experiments were done per silicon wafer diagonally, with two near the edge and one in the middle, with an ion dose of 5.65 × 10^11^ ions/cm^2^. Three 5 × 5 pixel areas were selected diagonally from each 32 × 32-pixel ToF-SIMS data tile, yielding 18 data points per sample type. This sampling method was used to minimise potential analysis artefacts. To compare the sensitivity of the beam to azoxystrobin with different cluster sizes, two major ion signals characteristic of AZ were normalised to the primary ion dose for five sputter cycles of analysis and were plotted and compared.

### ToF-SIMS imaging and depth profiling

The penetration of AZ through wheat leaves was probed using both depth profiling and imaging capabilities of ToF-SIMS. The optimised analysis conditions of the water cluster primary ion beam, tuned to 15,000 cluster size and an ion dose of 1.5 × 10^12^ ions/cm^2^/sputter cycle, were used for both depth profile and imaging studies. All ToF-SIMS data processing was done with the Analyse software (Ionoptika Ltd., Chandlers Ford, UK), Microsoft Excel, and Origin Pro (OriginLab Corporation, MA, USA) software.

For the depth profiling experiments, after the sample was fixed to the sample holder with the steel grid on top, it was taken to the in-built glove box of the ToF-SIMS instrument. The loaded sample holder was submerged in liquid nitrogen until fully frozen and transferred into the load-lock and pumped to 10^–5^ mbar pressure. The sample was dried overnight in the prep chamber of the instrument with a vacuum of < 10^–6^ mbar pressure for the sample stabilisation. An area of 320 × 320 µm^2^ on the leaf was randomly selected for the experiment, and the analysis was set up for 1000 sputter cycles.

In the imaging SIMS experiments, the leaf sample showed very little sample charging effect, as it was on a conducting tape. Appropriate sample bias voltage was applied during imaging experiments. The ToF-SIMS images were acquired with a spatial resolution/spot size of 7 µm.

### MALDI imaging

The MALDI imaging study was conducted using a Bruker RapifleX MALDI Tissuetyper™ fitted with an Nd:YAG laser (355 nm) in reflectron mode. After freeze-drying of the samples mentioned in section “Sample preparation of wheat leaves”, the leaf cryo-sections were sublimated with α-cyano-4-hydroxycinnamic acid (CHCA) using a Shimadzu iMLayer matrix vapour deposition system. The CHCA matrix was selected as found appropriate in previous studies with wheat leaves and azoxystrobin by Ikuta et al. [16]. For the delicate wheat leaf sample we used in our study, matrix sublimation was more suitable for maintaining the intact structure of the leaf and caused minimal artefacts compared to conventional matrix spraying methods. The matrix was deposited to an approximate thickness of 0.5 µm at 250 °C temperature. Red phosphorous crystals dissolved in methanol were used to mass calibrate the MALDI-TOF. The imaging was performed in a mass range of *m*/*z* 100 to 1200 in the reflectron mode with the Smartbeam parameter set to single. The imaging was done with a raster size of 20 µm with a scan size of 16 µm. The spectra were collected from 300 laser shots at 10 kHz frequency with a sampling rate of 1.25 GS s^–1^. The MALDI images were analysed using the Bruker SciLs Lab Pro software.

### Cryo-SEM of wheat leaves

The cryo-SEM experiments were conducted on the Hitachi SU8220 field emission scanning electron microscope. To perform the experiments, fresh leaf tissue of an approximate length of 1 cm was excised and mounted on a Quorum specimen shuttle with colloidal graphite mixed with Tissue-Tek® (O.C.T compound). The sample was plunge-frozen in liquid nitrogen slush before transferring to the Quorum PP3010T preparation chamber. The specimen holder was then transferred to a nitrogen-cooled sample stage in vacuum at − 140 °C, and was freeze-fractured if the leaf cross-section was imaged using a clamp specimen holder. The sample was sublimated at − 95 °C for 5 min before coating with gold/palladium for 30 s with a current of 5 mA and a temperature of − 120 °C. Post-coating, the sample was transferred to the SEM cooled stage at −160 °C for secondary or backscattered electron images.

## Results and discussion

Plants have not yet been extensively studied as a model for depth profile experiments using ToF-SIMS. In this study, we developed a workflow that integrates the depth profiling and mass spectrometry imaging capabilities of ToF-SIMS to investigate the movement of a commercial azoxystrobin (AZ) formulation through the surface layers and bulk tissue of wheat leaves. We conducted thorough optimisation of the ToF-SIMS analytical conditions to enhance the sensitivity for azoxystrobin, and these optimised settings were subsequently used in our experiments. The AZ formulation was applied to the plants, with sampling done at 24 h and 1 week post-application, allowing us to observe the *in-planta* mobility of azoxystrobin in wheat leaves.

### Optimising ToF-SIMS conditions for azoxystrobin formulation

**Fig. 1:**
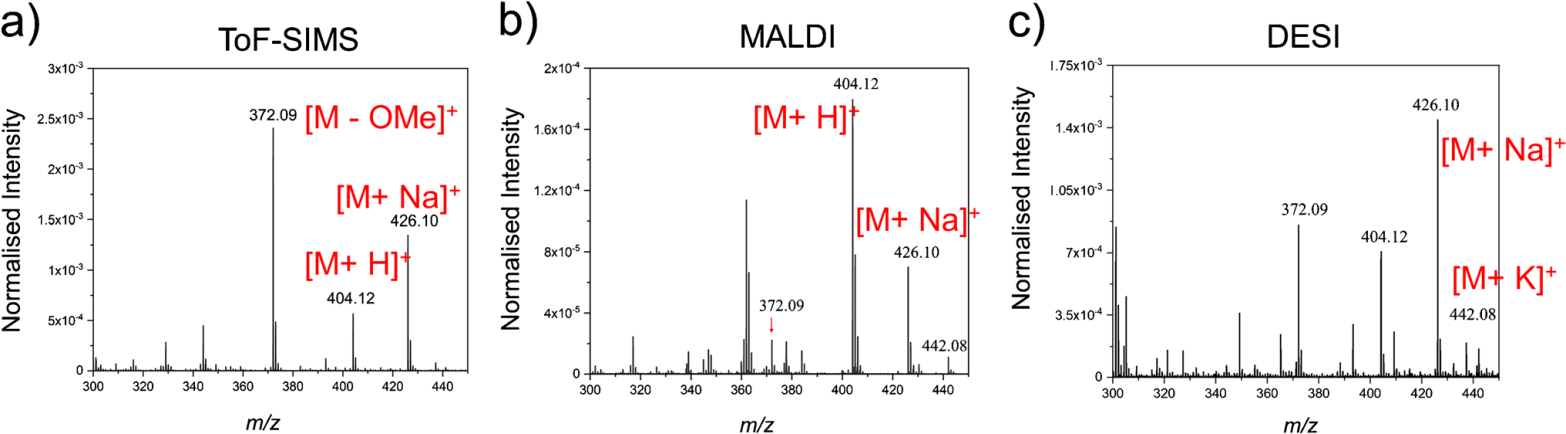
Comparing azoxystrobin formulation spectra between different mass spectrometry techniques. **a** ToF-SIMS spectrum acquired with the (GCIB-H2O)_15k_ ion beam, **b** MALDI spectrum, and **c** DESI spectrum. All spectra were normalised to the total ion count for comparison

**Fig. 2:**
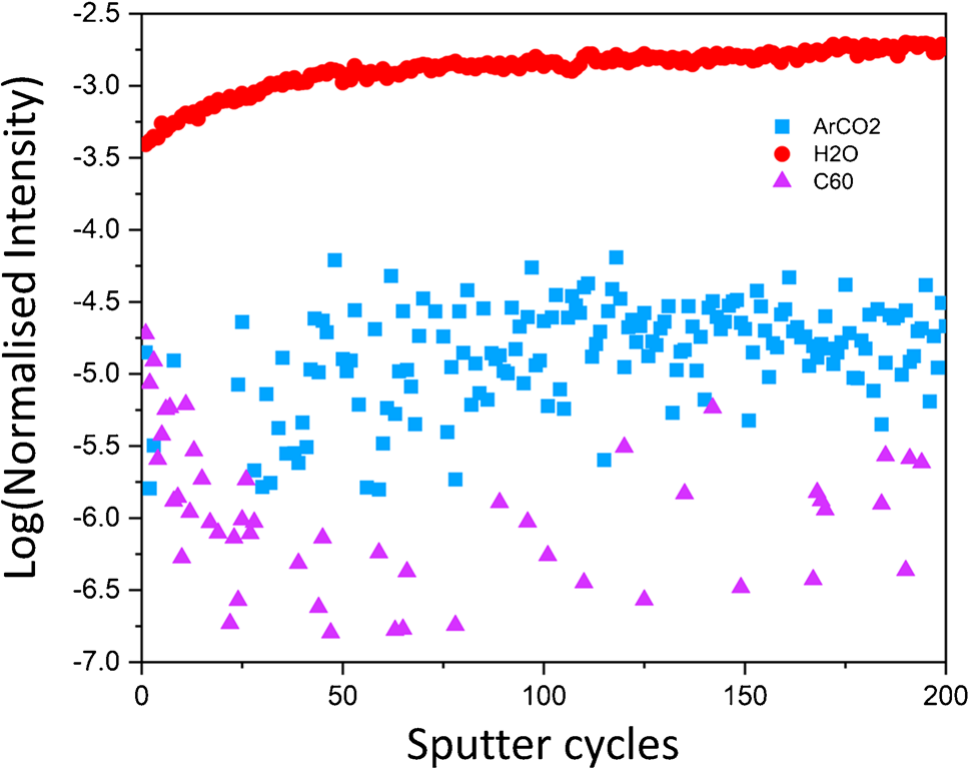
Comparing depth profile efficiency of different primary ion beams. The GCIB-H_2_O(red), GCIB-Ar/CO_2_ (blue), and C_60_^+^(purple) ion beams were used for a 200 sputter cycle depth profile on vacuum-dried wheat leaf applied with azoxystrobin (*m*/*z* 372.1). The comparison of the log of TIC normalised depth profile data for the three beams showed significantly enhanced sensitivity of the GCIB-H2O beam

In ToF-SIMS, unlike softer ionisation methods like DESI and MALDI, the mass spectra often show the molecular ion and several of the major fragments (see Electronic Supplementary Material, Table S1). The difference in the AZ formulation mass spectrum obtained with ToF-SIMS, DESI, and MALDI is shown in Fig. 1. The primary ion beams available to us for analysis with ToF-SIMS, C_60_^+^, GCIB-H_2_O, and GCIB-Ar/CO_2_ were tested to understand the variation in sensitivity to azoxystrobin in the wheat leaf matrix. A vacuum-dried leaf treated with AZ formulation was depth profiled using different primary ion beams for 200 sputter cycles of data acquisitions using the same ion dose and similar acquisition conditions. The data acquired was normalised by total ion count per sputter cycle and log-scaled. The comparison of the data revealed a much greater sensitivity for the AZ [M-OMe]^+^ ion, *m*/*z* 372.1, for the GCIB-H_2_O beam, followed by the GCIB-Ar/CO_2_ beam and the C_60_^+^ ion beam in the wheat leaf (Fig. 2). The trend observed was expected and in accordance with several previous reports showing that the GCIB-H_2_O beam has elevated sensitivity for bio-organics in comparison to other GCIB and other primary ion beams [21, 22].

**Fig. 3:**
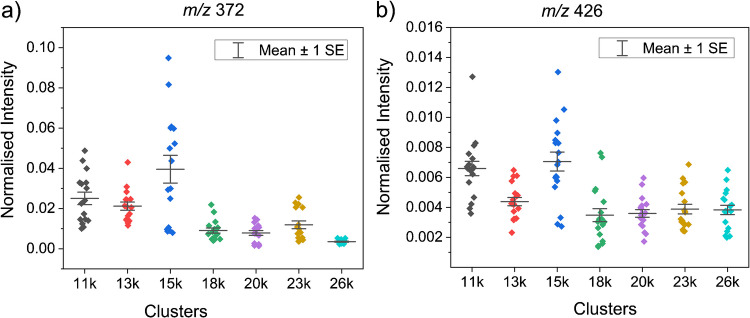
Sensitivity test for various cluster sizes. The normalised intensity obtained for azoxystrobin ion signals (*m*/*z* 372.1, *m*/*z* 426.1) for water cluster beams with varying energy per unit mass or cluster sizes was compared. For all clusters, the mean and the standard error are shown. For both *m*/*z* 372.1 (**a**) and *m*/*z* 426.1 (**b**), the 15 k cluster size shows elevated sensitivity

GCIBs can produce a diverse range of cluster sizes based on energy per unit mass, which significantly affects analyte sensitivity [23]. Thus, a test was conducted to compare the ToF-SIMS sensitivity for azoxystrobin formulation on silicon wafers. The base peak from azoxystrobin (*m*/*z* 372.1) displayed an increased normalised intensity at a cluster size of 15 k with an energy per unit mass of 0.25 eV/u (Fig. 3). A similar distribution pattern was observed when comparing the normalised intensity of the sodium adduct of azoxystrobin. Hence, a water cluster ion beam with a cluster size of 15 k was chosen for further experiments with azoxystrobin.

**Fig. 4:**
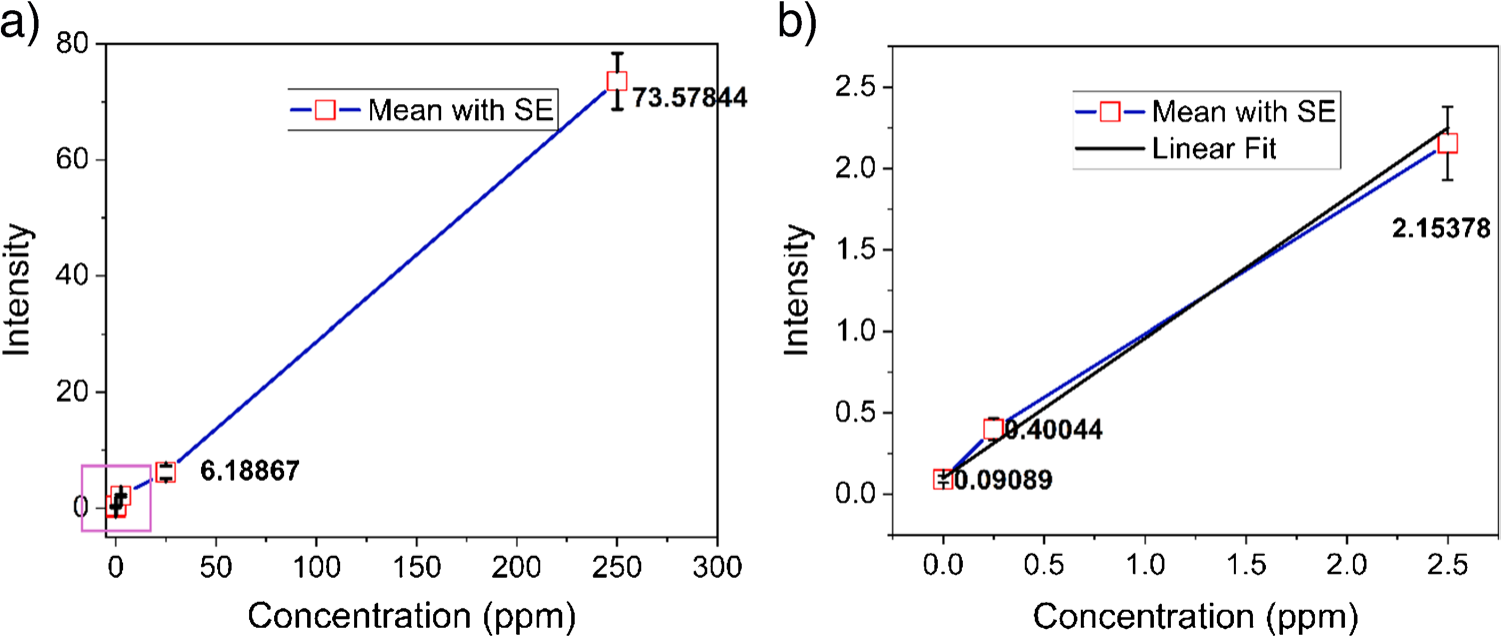
Limit of detection. A tenfold dilution series experiment was conducted on azoxystrobin formulation drop-casted and air-dried on silicon wafers from 250 to 0.25 ppm. Intensity per pixel was plotted and compared with 18 data points per concentration. The data points in the purple square in **a** are expanded and shown in **b**. A linear fit of the data (black line) points in **b** was used to calculate the limit of detection, which was seen to be nearly 0.25 ppm, as the data showed a more linear trend in the lower concentrations

To determine the limit of detection of AZ formulation, a ten-fold dilution study with azoxystrobin formulation from 250 to 0.25 ppm was conducted (Fig. 4). Since the lower concentrations of azoxystrobin showed a linear relation, they were used to determine the limit of detection. A linear fit of the intensity per pixel for 2.5 ppm, 0.25 ppm, and blank samples combined with an assumption of signal-to-noise of three for the limit of detection revealed a concentration of nearly 0.25 ppm as the possible limit. A two-sample *t*-test was done to detect how the intensities varied for these adjacent concentrations in the tenfold dilution test. As seen in Figure S1, all the concentrations had significant differences in comparison to the immediate neighbour (*p* < 0.05), revealing the high sensitivity of ToF-SIMS for agrochemical analysis.

### Elucidating depth penetration of azoxystrobin in wheat leaves

ToF-SIMS analysis of leaves for mapping metabolite or xenobiotic distributions requires two critical steps: (1) preserving the sample’s original chemical architecture, and (2) ensuring sample stability under the instrument’s high vacuum environment. These steps are essential for accurate in situ chemical characterisation. A cross-section of a leaf can be imaged by appropriate cryo-sectioning methods and storage, followed by analysis in the freeze-dried state. Depth profiling of whole leaves is relatively unexplored owing to the challenges in stabilising the sample and navigating the uneven, heterogeneous leaf surface. This study conducted comprehensive experiments to develop and optimise leaf sample preparation protocols for ToF-SIMS analysis, focusing on preserving the native chemical composition of the specimens.

**Fig. 5:**
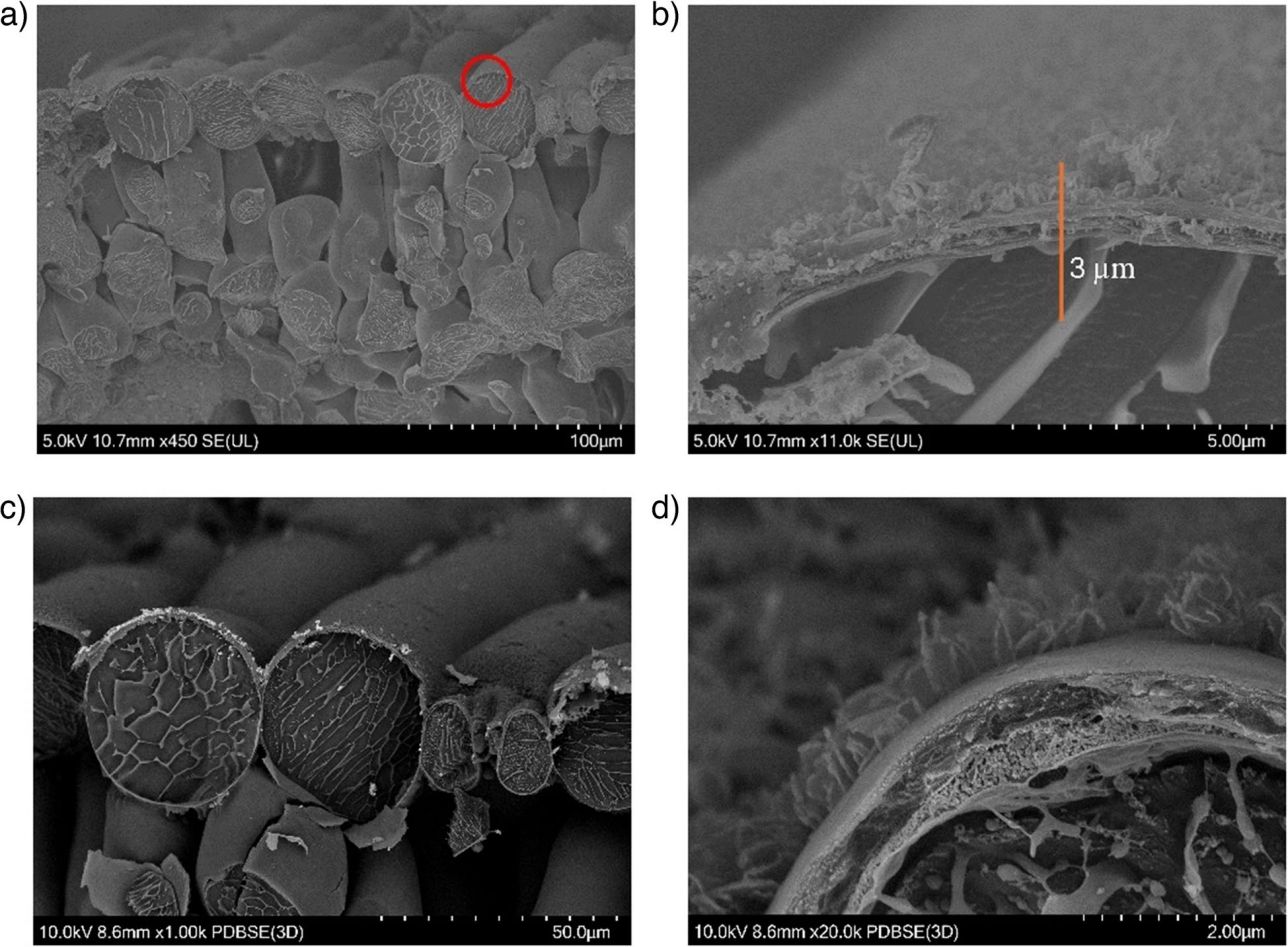
Cryo-SEM images of wheat leaf cross-section. **a** Secondary electron mode image of a cross-section of wheat leaves showing the surface of the leaf, epidermal cells, and the inside mesophyll cells. The red marked area in **a** is the zoomed-in area shown in **b**, showing the surface wax crystals, further intracuticular wax layer, cell wall, and the inside of the cell. The inset line in **b** is the suspected depth that is reached in 1000 sputter cycles of depth profiling. **c**, **d** Polarised diffused backscattered electron (PDBSE) mode images of wheat leaves. In **c**, the cross-section and surface of the leaf can be seen, and in **d** is a zoomed-in image of the surface layers

The surface of the wheat leaf is heterogeneous, with ridges and trichomes protruding (see Electronic Supplementary Material, Figure S2). When applied, an agrochemical must traverse a variety of chemically and topographically uneven layers to reach the bulk of the tissue. Wheat leaves have an intricate wax structure, starting with the epicuticular wax crystals on the outermost layer, followed by the waxes of the cuticle layer, as seen in Figure S3. The freeze-fractured cross-section of a leaf imaged with cryo-SEM is shown in. In, the wider cross-section of a leaf can be seen with the tightly packed epidermal cells, followed by the spongy and loosely packed mesophyll cells, within which a section of the vascular bundle can be seen. The inset red circle in is shown in an expanded view in with the epicuticular wax crystals, the cuticular layer, the cell wall, and the inside of a cell being observed. Figure 5c and d show the polar diffused backscattered electron (PDBSE) images showing the difference in atomic numbers in different cross-section regions. In PDBSE, the brighter the signal, the higher the atomic number and vice versa, hence showing contrast in the local chemical environment, including the hydrophobic cuticle and the hydrophilic cell wall and insides of the cell. Figure 5d illustrates the chemical complexity of the distinct layers from the leaf surface to the interior of the epidermal cells. The depth profile experiments aim to elucidate the chemical mobility of the applied agrochemical through these heterogeneous structures.

**Fig. 6:**
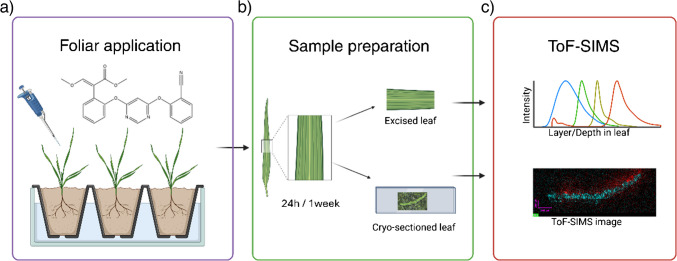
Workflow of experiments to understand the active penetration of azoxystrobin in wheat leaf. **a** Wheat plant leaves at 10 to 15 days, when the plant is typically at the two-leaf stage, are applied with 10 µL 2500 ppm azoxystrobin formulation in the middle of one of the two true leaves. **b** The area of application of the formulation is sampled at 24 h or 1 week after formulation application and is **c** either taken for direct tissue depth profile or for cryo-sectioning and SIMS imaging. Created in BioRender

**Fig. 7:**
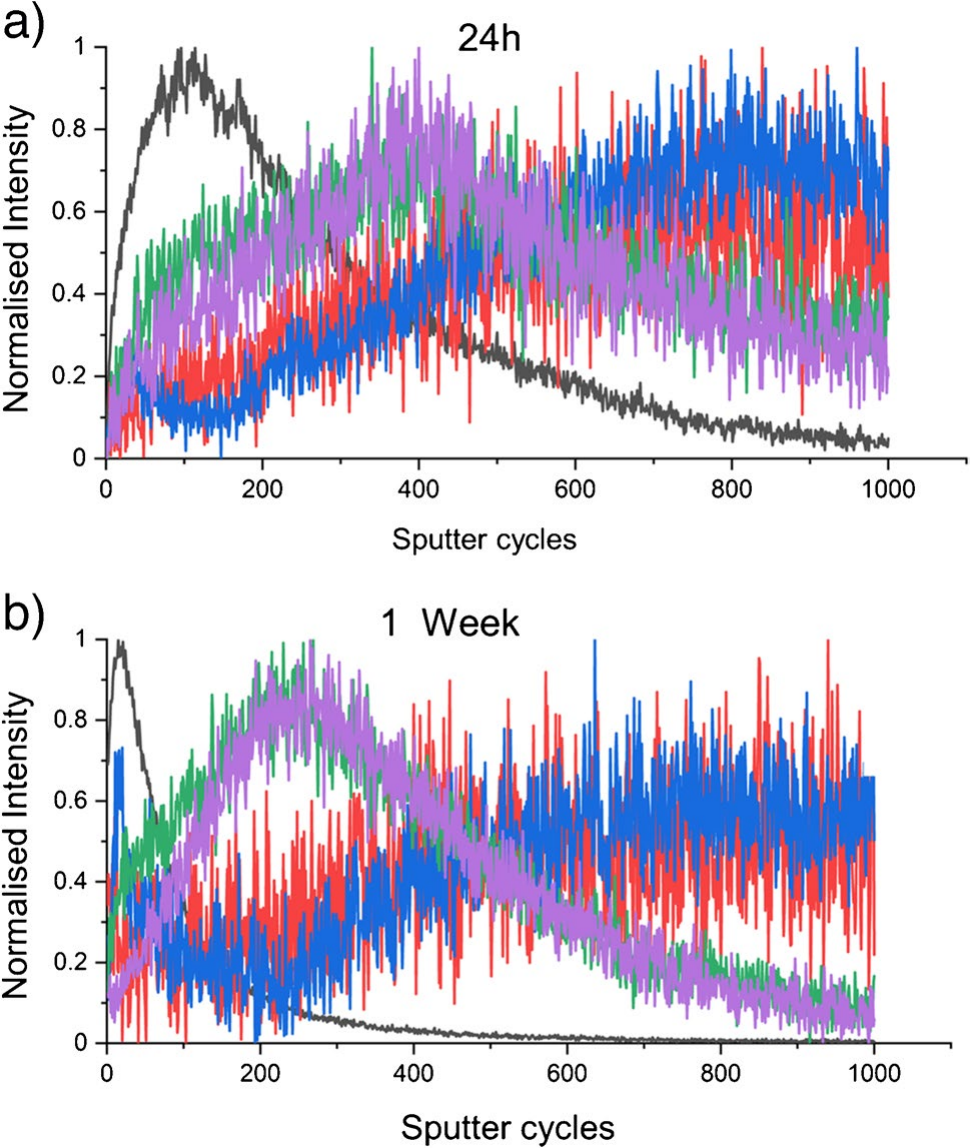
Depth profile data for wheat leaves applied with the azoxystrobin formulation at 24 h and 1 week. The leaves in healthy wheat plants were applied with 2500 ppm azoxystrobin formulation and were sampled after 24 h and 1 week of application. In the depth profile images shown through the 1000 sputter cycles of analysis, the black line is for the azoxystrobin base peak (*m*/*z* 372.1), green and purple are for cellulose markers (*m*/*z* 145.5 and *m*/*z* 127.04), and red and blue are for alanine and lysine, the bulk tissue markers (*m*/*z* 44.05 and 84.08)

The experimental workflow designed to investigate azoxystrobin penetration into wheat leaves is illustrated in Fig. 6. Analyses were conducted on 320 × 320 µm^2^ regions of interest. A depth profile analysis comprising 1000 sputter cycles revealed the distribution of secondary ion markers associated with tissue bulk and cell walls. This analysis suggests that our ablation and profiling encompassed the surface layers of wheat leaves, including the cuticular waxes and cell wall, extending into the underlying tissue. The depth profile analysis of chemical markers indicated that a 1000 sputter cycle examination reached roughly 3 μm into the leaf tissue (Fig. 5b). Depth resolutions of < 10 nm per sputter cycle could be achieved with GCIBs on a homogenous organic layer [24, 25]. A depth profile of 1000 sputter cycles done on a standard material like Irganox 1010 can yield similar depth penetration as estimated, of a few microns, as shown by Sano et al. [24]. However, due to the heterogeneity of different surface leaf layers, a careful calibration of depth resolution with respect to specific layer chemistries is required for a more accurate understanding of the depth of penetration into the different layers of the leaf. Comparison of the azoxystrobin signal with diagnostic secondary ion markers for cellulose and amino acids revealed a continuous translocation of azoxystrobin through the surface layers into the cellular compartments and leaf bulk. This movement was evident at 24 h post-application and persisted for at least 1 week (Fig. 7). These findings align with previous studies on azoxystrobin mobility, which have demonstrated that the compound sticks to the cuticular waxes, forming a reservoir that facilitates gradual penetration into the leaf tissue over several weeks [16]. The heterogeneous surface morphology of wheat leaves introduces significant variability in ion signal distribution across samples. Still, the relative distribution pattern of the AZ formulation in relation to tissue markers remains consistent, as evidenced by the three replicates of the 24-h depth profile experiment (see Electronic Supplementary Material, Figure S4). Multiple sample preparation methods were evaluated for depth profiling experiments (see Electronic Supplementary Material, Supplementary Note 1 and Figure S5). The protocol described in section “ToF-SIMS imaging and depth profiling” was ultimately selected based on the quality of data obtained and the relative ease of sample preparation. Notably, markers for leaf wax layers are more readily detectable in negative ion mode, as illustrated in Figure S6.

### MSI of wheat leaf cryo-sections

**Fig. 8:**
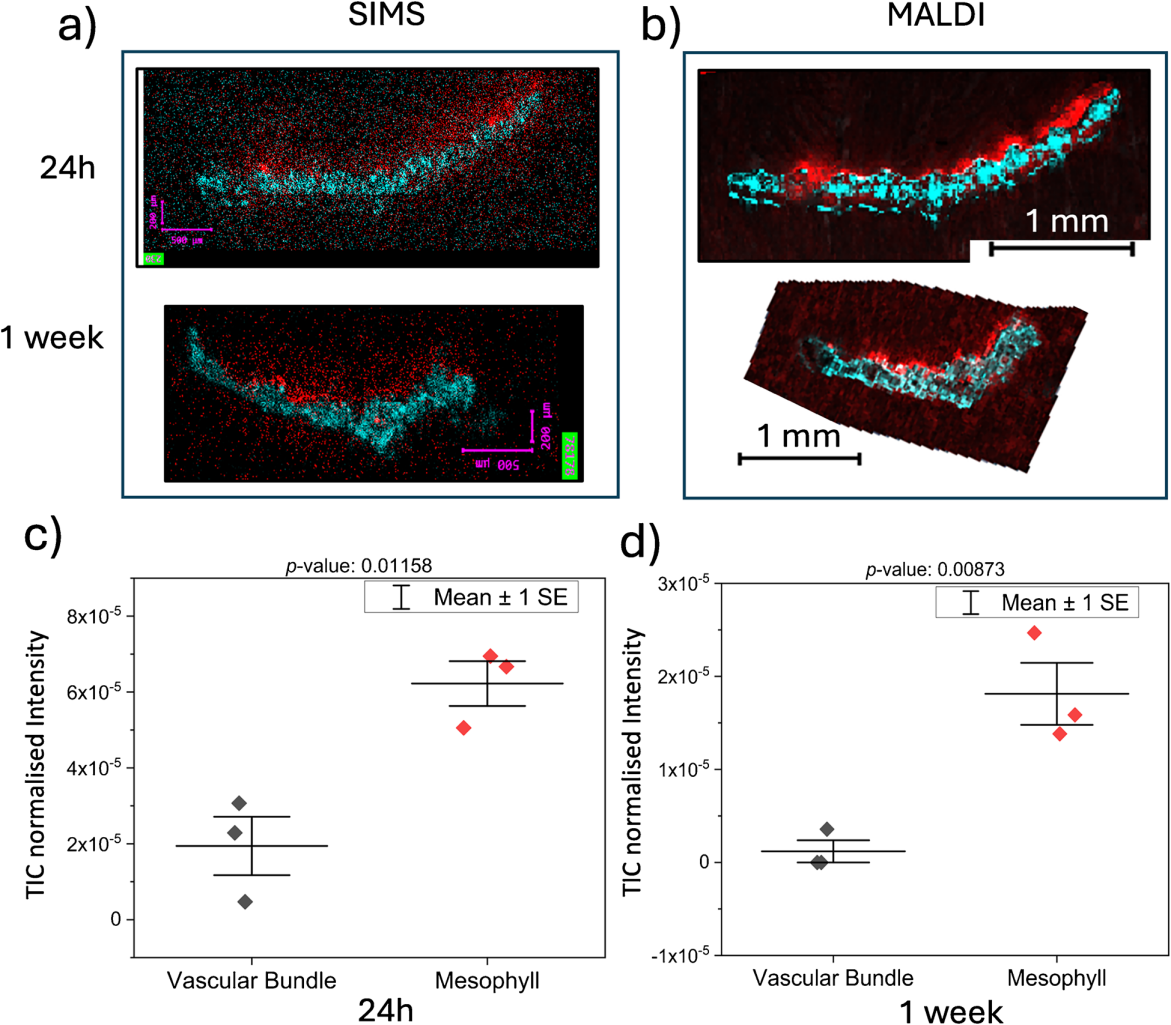
Cross-section images of wheat leaves with ToF-SIMS and MALDI. **a** Parallel wheat leaf sections of the same leaf sample were imaged with ToF-SIMS and **b** MALDI after similar sample prep for comparison. In the ToF-SIMS image, the red signal is for azoxystrobin (*m*/*z* 372.1) and the cyan signal is for lysine (*m*/*z* 84.1). In the MALDI image, the red signal is for azoxystrobin (*m*/*z* 442.1) and the cyan signal is an unidentified peak from the leaf at *m*/*z* 492.1. A two-sample *t*-test comparison of the ToF-SIMS intensities obtained for azoxystrobin from the vascular bundle and mesophyll regions of interest selections over several pixels is shown in **c** and **d**. An increased intensity of azoxystrobin is observed in the mesophyll compared to the vascular tissue at 24 h and 1 week, respectively, with a more pronounced difference at 1 week

Depth profiling provides comprehensive insights into the translocation of agrochemicals through surface layers within a selected region of interest. Imaging the entire cross-section of leaf samples reveals the spatial distribution and penetration patterns of applied agrochemicals at high resolution, allowing for pixel-by-pixel analysis. In such heterogeneous samples as plant tissues, this provides potentially greater detail in distribution, which might be missed when averaging signals over several pixels during a depth profile. Young wheat leaves are quite fragile, needing extreme care when handling to maintain the innate structure during analysis. The process of cryo-sectioning helps maintain a pristine structure along with the innate chemical localisations. The traditional cryo-sectioning method of snap freezing of leaves, sectioning, and thaw-mounting was ineffective for the tender leaves used in this study. Hence, a specialised embedding and cryo-sectioning method was developed after much optimisation (see Electronic Supplementary Material, Supplementary Note 2, Figure S7). We observed that the chemical leakage was lower and the sample quality was higher if the sample was cryo-sectioned and stored in a −80°C freezer within a few hours of embedding in CMC (see Electronic Supplementary Material, Supplementary note 2). Wheat leaf cryo-section samples at 24 h and 1 week after treatment were imaged with the 15 k water cluster ion beam at a spatial resolution of 7 to 8 µm with ToF-SIMS. At 24 h, most of the AZ ion signal is seen on the adaxial surface of the treated leaf with little distribution seen in the bulk of the leaf (Fig. 8a). One week after formulation application, the fungicide concentration increased significantly in the inner mesophyll cells near the vascular bundles. Comparisons from regions of interest revealed an increased presence of the AZ intensity in the mesophyll of the leaf cross-sections than in the vascular bundle (Fig. 8c and d). A two-sample *t*-test of the AZ intensities in the two regions in the two cross-sections gave a lower *p-*value for the 1 week, showing more pronounced differences. Therefore, more penetration of AZ into the bulk of the tissue was observed by 1 week compared to the 24-h sample. The relatively low intensity of AZ in the vascular tissue area in the leaf might be due to the active xylem transport of AZ when reaching the vascular tissue [16]. The MALDI analysis of parallel sections of the sample showed a similar distribution but at a lower spatial resolution due to the pixel size limitations of the technique. The bulk penetration and fungicide distribution observed are in agreement with a previous MSI study by Ikuta et al. [16].

## Conclusion

Leaves present unique challenges for ToF-SIMS analysis due to their delicate structure and heterogeneous composition. The lack of previous literature on this subject underscores the complexity of analysing such unconventional samples using this technique. Traditional ToF-SIMS analytical and sample preparation strategies are not very useful when attempting to analyse the intact chemical distribution in a leaf. Herein, we have described a comprehensive experimental methodology to test, optimise, image, and depth profile the *in-planta* movement of an applied agrochemical in wheat plants. Azoxystrobin, a widely used fungicide, was selected for this study. After foliar application, this active ingredient moves into the bulk of the tissue and acts as a chemical barrier between the plant and the fungal pathogen. Comparative analysis of various ToF-SIMS primary ion beams revealed superior sensitivity to azoxystrobin when using the water cluster ion beam, as opposed to the argon-carbon dioxide cluster or C60^+^ ion beam. The energy per unit mass (velocity) of ion beams significantly influences analyte sensitivity. The systematic evaluation demonstrated that a water cluster ion beam with a size of 15 k provided optimal sensitivity for azoxystrobin among the tested cluster size range. Utilising the optimised 15 k water cluster ion beam, analysis of serially diluted azoxystrobin formulations yielded a limit of detection (LOD) of 0.25 ppm for azoxystrobin with the ToF-SIMS instrument used.

To understand the movement of a foliar-applied agrochemical on wheat, depth profiling and imaging modes of ToF-SIMS analysis were applied with the optimised beam conditions. The depth profile experiments gave a novel overview of how azoxystrobin permeates the chemically heterogeneous nanometre-scale surface layers of a leaf. Cross-section imaging allows the monitoring of agrochemical penetration in the leaf bulk at a pixel-by-pixel scale. For both types of experiments, ToF-SIMS was used to test and optimise various sample preparation methodologies, a vital step in understanding the true *in-planta* distribution of an innate metabolite or xenobiotic. The high-water content and fragility of wheat leaves required the use of a specialised cryo-sectioning methodology using mass spectrometry-compatible Kawamoto tapes. We observed a continuous movement of azoxystrobin through the surface layers of the leaf, even at 24 h with the depth profiling experiments, which persisted at 1 week after formulation application. When considering the cross-section images obtained at the same time point, an increased distribution in the mesophyll tissue at 1 week was observed compared to that at 24 h. In both time points, the relative distribution in the mesophyll was increased compared to the vascular tissue, probably due to the active xylem transport of azoxystrobin. The chemical distribution data obtained from cryo-sectioned samples via ToF-SIMS imaging were corroborated by complementary MALDI-MSI analysis. Furthermore, cryo-SEM imaging of analogous samples enabled the correlation of observed chemical patterns with specific leaf morphological features. Our results are in accordance with previous reports on azoxystrobin mobility. We believe that the optimised ToF-SIMS analytical and sample preparation techniques presented here can be applied to any agrochemical-foliar system, providing valuable insights into the penetration and movement of agrochemicals. ToF-SIMS provides unique data concerning the intricacies of agrochemical movement through the highly heterogeneous surface layers, crucial in dictating the efficacy of an applied agrochemical in plants. By adopting this ToF-SIMS workflow, researchers can gain a more thorough understanding of how foliar-applied agrochemical formulations behave. Incorporating these methods into the research and development processes within the agrochemical industry can help in the creation of safer and more rigorously tested agrochemicals, ultimately contributing to the development of sustainable solutions to address global food security challenges.

## Supplementary Information

Below is the link to the electronic supplementary material.

Supplementary Material 1 (DOCX 3.95 MB)

## Data Availability

The data used in support of this study is available on request from the corresponding author.
