# Supplementary material for: Resolving agrochemical penetration in wheat leaves with secondary ion mass spectrometry imaging and depth profiling
Source: Anal Bioanal Chem. 2025 Sep 30;417(28):6427–37. doi: 10.1007/s00216-025-06134-1 (PMC12596295; doi:10.1007/s00216-025-06134-1)
Supplement: Supplementary file 1 — Supplementary Material 1 (DOCX 3.95 MB) [file 216_2025_6134_MOESM1_ESM.docx]

Supplementary Information for:

**Resolving agrochemical penetration in wheat leaves with secondary ion mass spectrometry imaging and depth profiling**

Akhila Ajith,^1^ Sadia Sheraz,^1^ Aline Xavier de Souza,^2^ Drupad K Trivedi,^3^ Jean-Yves Mugnier^2^

Giles N Johnson,^4^ Phillip J Milnes,^2^ Nicholas P Lockyer^1*^

*^1^Photon Science Institute, Department of Chemistry, University of Manchester, Manchester, UK. E-mail:*[*nick.lockyer@manchester.ac.uk*](mailto:nick.lockyer@manchester.ac.uk)
*^2^Syngenta, Jealott's Hill International Research Centre, Bracknell, UK*
*^3^Manchester Institute of Biotechnology, Department of Chemistry, University of Manchester, Manchester, UK*
*^4^Department of Earth and Environmental Sciences, University of Manchester, Manchester, UK*

**Supplementary notes**

**Note 1: Comparing sample preparation strategies for depth profile**

To understand the effect of sample preparation conditions on the data obtained with depth profiling, several sample preparation conditions were tested. All the plants were treated similarly by droplet application of 10 µL of 2500 ppm azoxystrobin formulation on one of the two true leaves of the wheat plant. The plants were then uprooted and taken to the lab, maintaining pristine conditions. The area of application was then precisely excised using a clean single-edge blade and was stuck to the SIMS sample stub using double-sided carbon tape. Since the leaves are thick and insulating, a stainless-steel grid was placed on top of the leaf sample to help with sample charging. Stabilising the sample in the high vacuum of the ToF-SIMS instrument is essential for obtaining reliable information on the penetration of the agrochemical in the leaf sample.

To obtain sample stabilisation in the instrument, we tried three strategies and compared how the data obtained looked for azoxystrobin concerning the markers for different layers. Firstly, we kept the leaf sample in the prep chamber of the instrument overnight to dry and stabilise it and then analysed it in the sample analysis chamber (SAC) the next day. Secondly, we snap-froze the stub with the leaf sample and grid on top in liquid nitrogen in the glove box of the ToF-SIMS instrument and quickly transferred the sample to the high vacuum of the prep chamber and left it to stabilise overnight to create a freeze-dried sample. Lastly, we snap-froze the sample stub with the sample and grid on top in liquid nitrogen and also cooled the analysis chamber of the instrument to around -150 ˚C with liquid nitrogen and analysed the sample frozen hydrated. This sample preparation was the most labour and cost-intensive, with around 50 L of liquid nitrogen required for one day of analysis, and there were local restrictions due to health and safety concerns in cooling the instrument with liquid nitrogen overnight. Frozen hydrated experiments help provide the most authentic chemical distribution in a sample, but the formation of ice on top of the sample can be a possible artefact in such experiments. Multiple experiments were conducted for all three different sample preparation conditions, and it was concluded from the different experiments that all of them provided similar results for our research question and showed that freeze-drying did not introduce significant artefacts in our region of interest of the sample (Figure S5). Hence, the freeze-dried sample preparation was selected for further experiments and interpretation, considering the quality of the sample and ease of sample preparation.

**Note 2: Optimising mass spectrometry imaging sample preparation**

The careful handling and preparation of young wheat leaves for cryo-sectioning and imaging are vital to understanding the accurate chemical information about agrochemical mobility in plant leaves. Cross-sectioning a leaf to reveal the inner composition is a method compatible with high spatial resolution MSI to accurately image the intricate structural and chemical composition. Cross-section imaging of thin wheat leaves might not be well suited for techniques like DESI MSI if done at an imaging resolution of 50 μm or above, as the cross-section of a leaf itself is 200-300 μm in width, and interested features might be smaller than the spatial resolution.

Leaf cross-sectioning needs to be done at low enough temperatures to form small ice crystals and avoid the formation of large ice crystals to preserve the chemical and structural constitution of a leaf. Since plant leaves are small and fragile, the usual cryo-sectioning steps followed for animal tissues cannot be followed. In general, for animal tissues, the samples are preserved in iso-pentane at -80°°C for a significant amount of time and can be taken for cryo-sectioning when required. Since usually the size/thickness of the sample is very much larger than the size of the cutting blade used in a cryostat, the sample can be directly fixed to the cryostat with the glue-like OCT. After trimming the sample to reveal the entire surface area of interest, the sections could be cut and directly thaw-mounted onto a normal glass slide or an ITO-coated glass slide. In the case of plant leaves, the leaf needs to be embedded in a suitable embedding medium below -80°°C (dry ice + hexane slurry) to provide the bulk sample for the blade to cut through easily. It was observed that the leaf seemed to disintegrate when thaw-mounted directly onto plain glass slides or ITO-coated glass slides. Therefore, to prevent this disintegration, the sections need to be cut directly onto a mass spectrometer-compatible tape (Kawamoto tapes, Section-lab, Japan) to preserve the chemical structure. The embedding of leaves for cryo-sectioning should be done in a medium which remains liquid at room temperature and solid at -80°°C to hold the sample together for sectioning. Since the media will be encasing the entire sample, it should be such that it gives minimal mass spectral interference and shouldn’t have very intense signals in the mass spectra obtained. From the literature survey, it was found that the popular embedding media for plant samples are various concentrations of CMC and gelatine[1-3]. To understand what the best workflow for imaging wheat leaves is, cross-sections were produced by various sample prep methodologies and were analysed for comparison with ToF-SIMS.

Different conditions were tested for the cryo-sectioning of plant leaves with 10-15-day-old wheat as a model plant. When we tested different matrices for embedding of leaf samples after snap-freezing, 4% CMC appeared to be a bit too thick a consistency to work with. The post-sectioning prep seemed more difficult with 4% CMC and the CMC ion signals coming from within the sections and droplets of CMC and water forming on the Kawamoto tapes when trying to desiccate (Figure S7). Also, the M1 matrix ( <1% CMC), when used, seemed to cause delocalisation of chemical signals (Figure S7). An intermediate CMC concentration of 2% CMC worked satisfactorily with minimal chemical delocalisation (Figure S7). After trying out several thicknesses of cross-sections, 20 μm thickness seemed to hold the leaf integrity together and gave the best morphology similar to the expected structure of cross-sections. 10% gelatin was not liquid in room temperature and hence was deemed unsuitable for embedding when done on a routine basis. Normal desiccation of plant leaf cryo-sections seemed challenging as the embedding media is a liquid at room temperature and when the temperature of the slide rises during desiccation, it causes the media to form droplets around and sometimes causes disintegration of the cross-section and chemical delocalisation.

As an alternative to normal desiccation, for better reproducibility of sample prep, we tried out freeze-drying, where the sample dries in cold conditions (-50 °C) hence decreasing the chance of sample damage.

In the J105 ToF-SIMS instrument, we have the option of doing multiple layers of imaging experiments on the same selected area, and it was observed that the total ion intensity as well as individual ion intensities tend to decrease as the layers of analysis increase. Hence, the first analysis of a sample was observed to give the best ion image of a sample (Figure S8).

**Table S1: *Common ion signals and Fragments of Azoxystrobin observed with ToF-SIMS***

| Ion​   (M = Azoxystrobin)  ​ | Theoretical *m/z*​ |
| --- | --- |
| [M+H]^+^​ | 404.1234​ |
| [M+Na]^+^​ | 426.1061​ |
| [M+K]^+^​ | 442.0081​ |
| [M-Me]^+^​ | 388.0928​ |
| [M-OMe]^+^​ | 372.0978​ |
| [M-COOMe]^+^​ | 344.1030​ |
| [M-Me-COOMe]+​ | 329.0795​ |

**Supplementary Images**

**
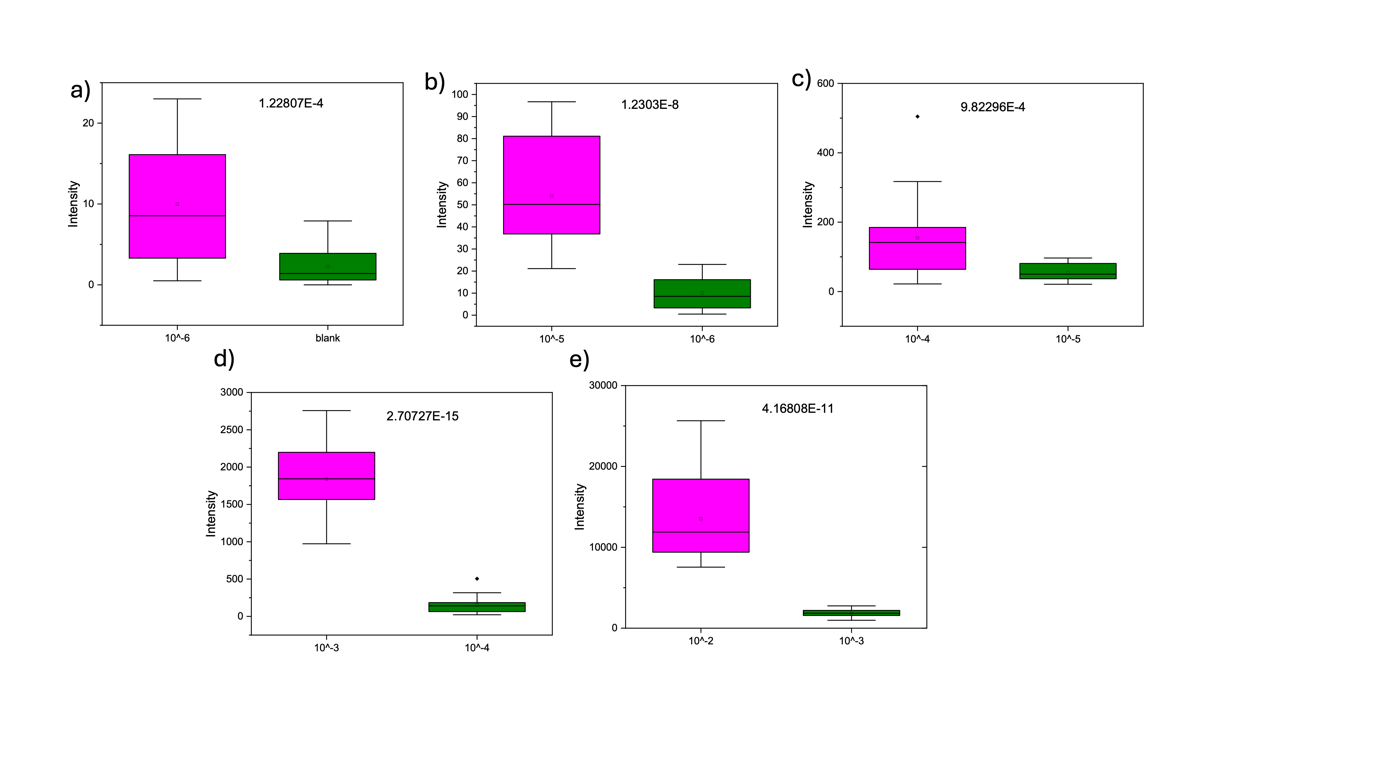
Figure S1**. Statistical comparison of adjacent concentration intensities in a ten-fold dilution series experiment with ToF-SIMS on azoxystrobin formulation (a-e). A two-sample t-test was used to compare the different concentrations. The x-axis lists the concentrations in ppm, and the y-axis shows the corresponding intensity distributions as a box plot. The inset number corresponds to the p-value in a two-sample t-test

**
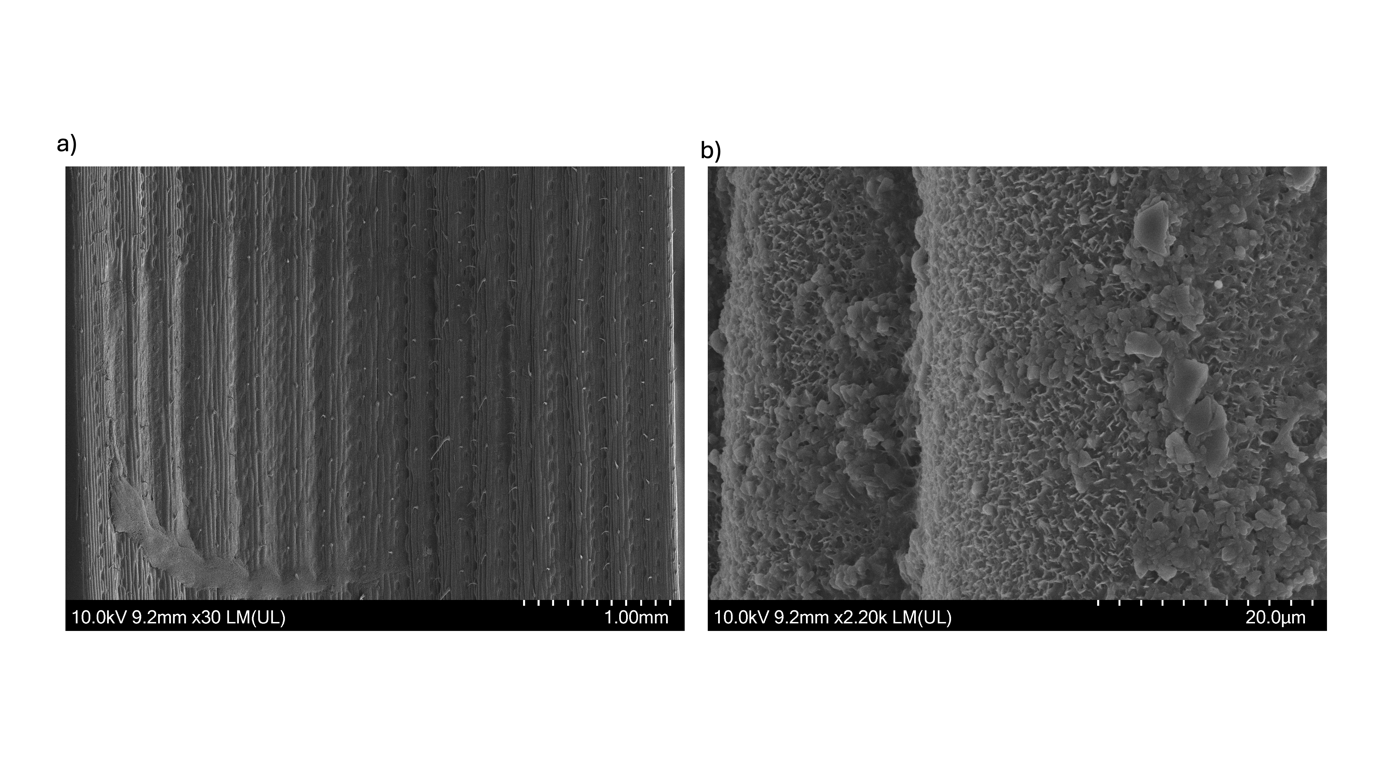
Figure S2**. Cryo-SEM images of a wheat leaf after application of the agrochemical formulation, with a) showing a wider view of the surface topology and b) showing a zoomed-in image of the ridges in the leaf. The fungicide formulation can be seen resting on the wax crystals of the leaf.

**
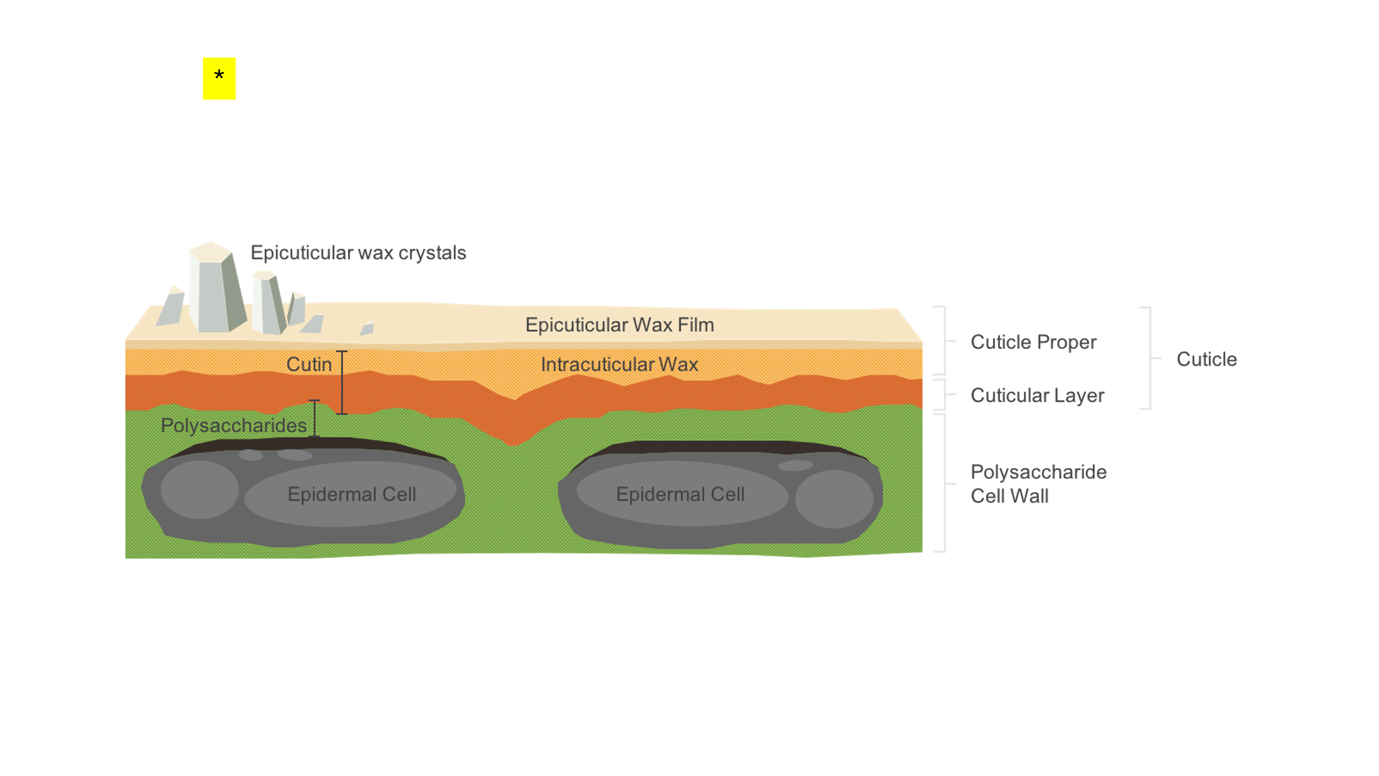
**

**Figure S3.** Pictorial representation of the upper layers of a leaf. Picture obtained from <https://www.cultiva.com/what-is-the-plant-cuticle/>

**
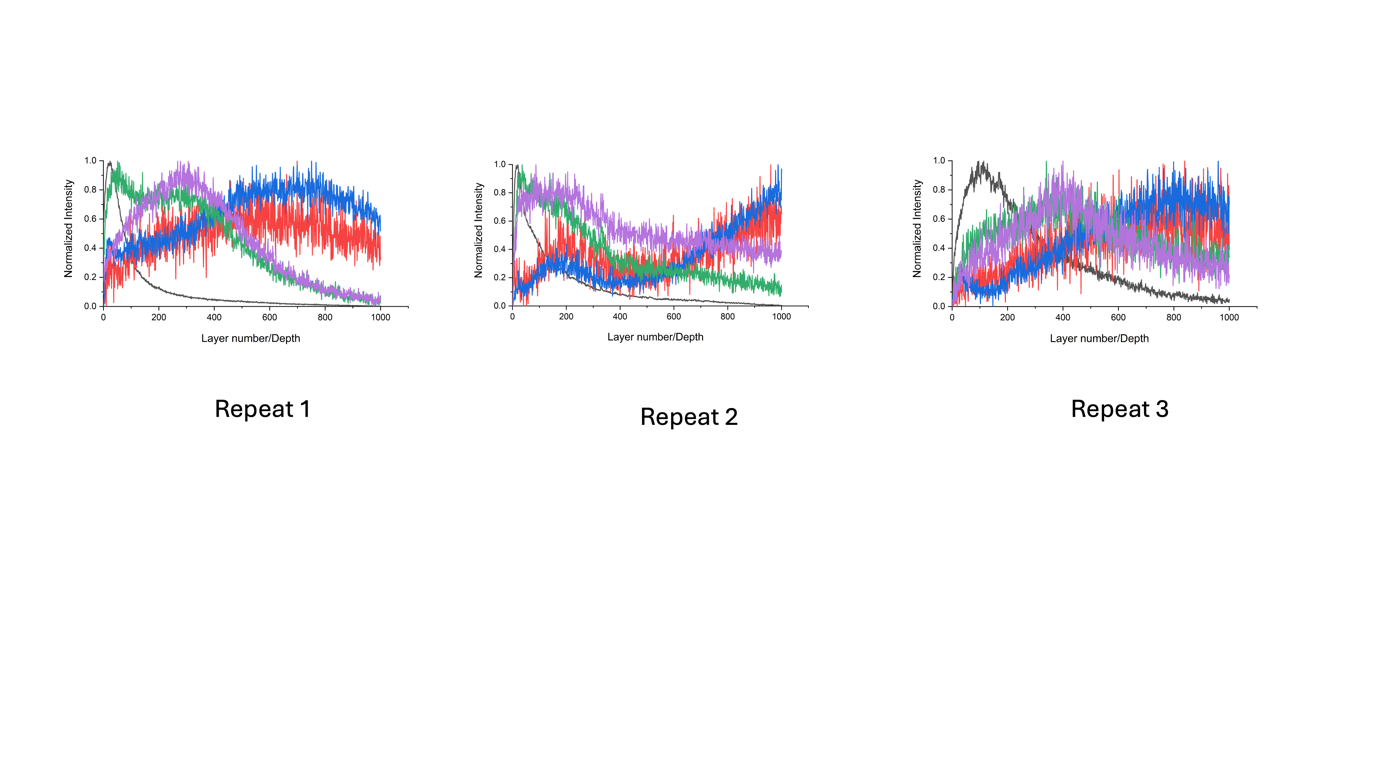
Figure S4**. Reproducibility of ToF-SIMS depth profile. The leaves were applied in-plant with a 2500 ppm azoxystrobin formulation and were sampled after 24h and 1 week after the formulation application. After the required time point was reached, the wheat plants were uprooted and brought to the analysis lab, maintaining pristine conditions. The area of interest for analysis was excised, snap-frozen in liquid nitrogen and dried in the instrument vacuum overnight for the sample to dry and stabilise. All samples were depth profiled for 1000 layers in an area of 320um^2^ with the GCIB-H_2_O 15k ion beam with an ion dose of 1.5e^12^/cm^2^/layer. In the depth profile images shown through the 1000 layers of analysis, the black line is for the azoxystrobin base peak (*m/z* 372.1), green and purple are for cellulose markers (*m/z* 145.5 & *m/z* 127.04) and red and blue are for alanine and lysine, respectively (*m/z* 44.05 & 84.08). Data were acquired on three different leaf samples.


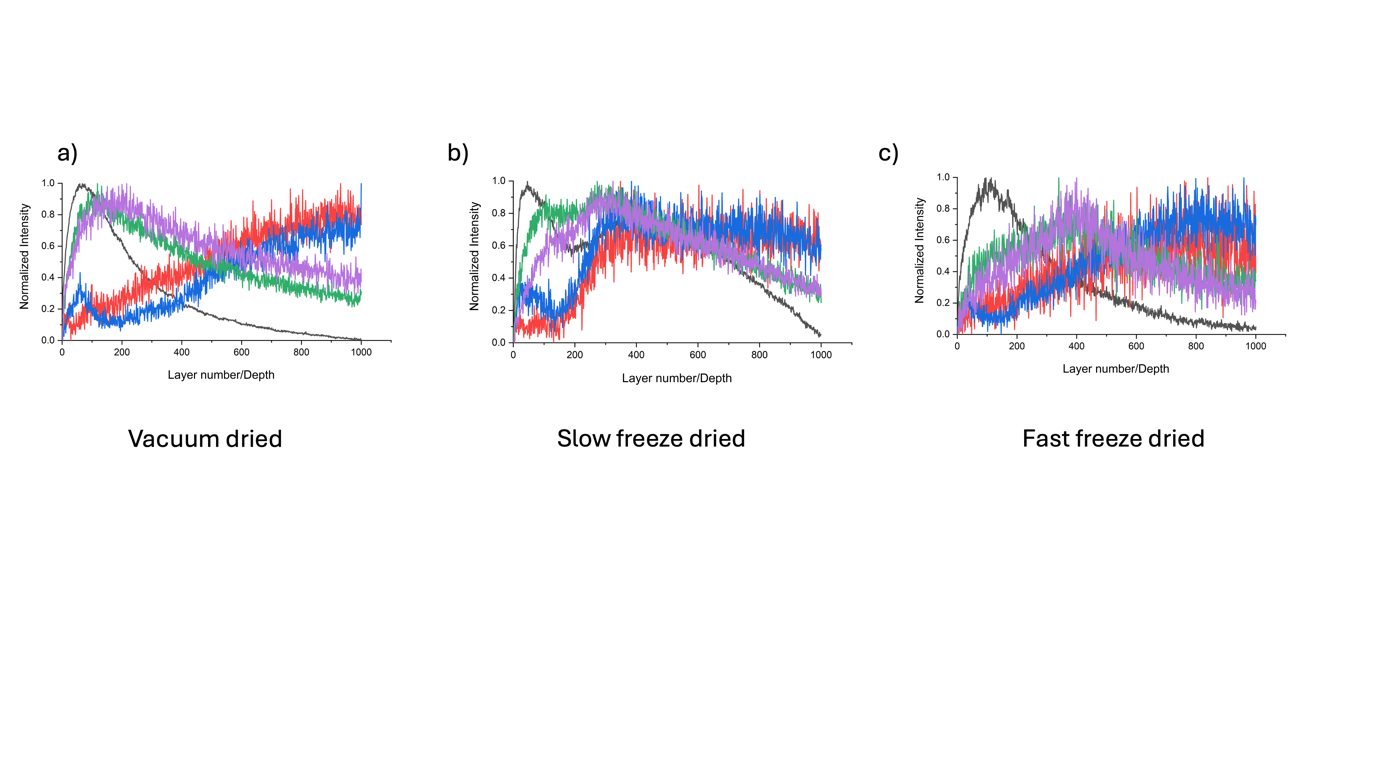


**Figure S5**. Comparing sample preparation methods for the azoxystrobin formulation applied to wheat leaves. All the above data were acquired on wheat leaves applied with azoxystrobin formulation and sampled after 24h.

**
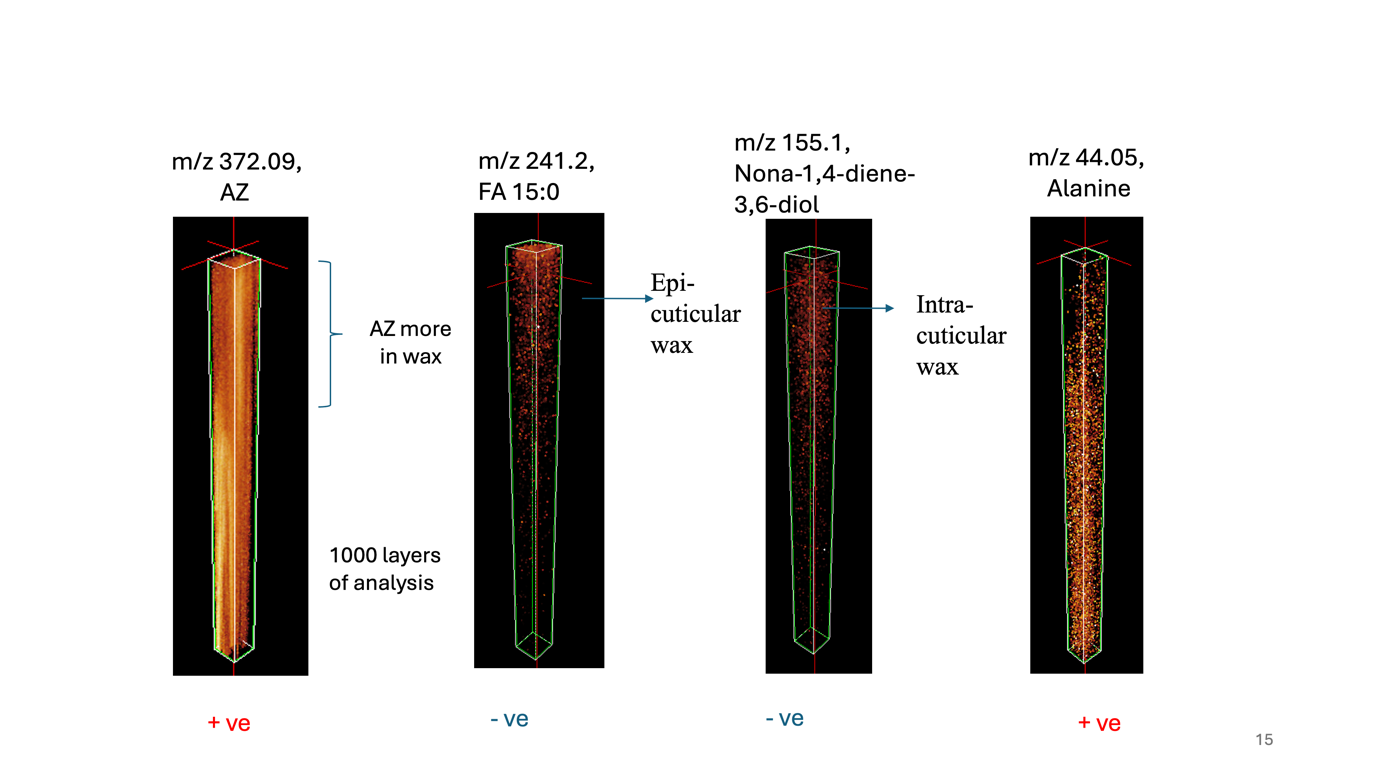
**

**Figure S6**. Visualising 3-D depth profile data (3-D Analyse software, Ionoptika Ltd). The depth profile data shown here is a 3-D representation of similar data as seen in Figure 4. This depth profile data was acquired on 10-15-day-old wheat leaves applied with 10 µL 2500 ppm azoxystrobin formulation, flash frozen and freeze-dried in an instrument vacuum. An area of 320 um^2^ was analysed for these experiments for 1000 layers in the positive and negative ion mode. The cuticular wax markers are mostly visible in negative ion mode, whereas the tissue bulk marker, *m/z* 44.05(alanine), is visible in positive ion mode.

**Figure S7**. Optimising cryo-sectioning conditions for wheat leaves after application of fungicide formulation. All leaves used for this test were between 10 to 15 days after planting the seeds in the two-leaf stage. All the images show the grey-scale ion image of m/z 184.07(phosphocholine headgroup) on samples prepared with different methods. a) 4% CMC embedded, stored overnight in -80˚C and post-sectioning desiccation in normal desiccator b) M1 matrix (<1% CMC, phenol, H2O) stored overnight in -80 C and post-sectioning desiccation in normal desiccator c) 2% CMC embedded leaves, sectioned within 4 hours and post-sectioning desiccation in normal desiccator.


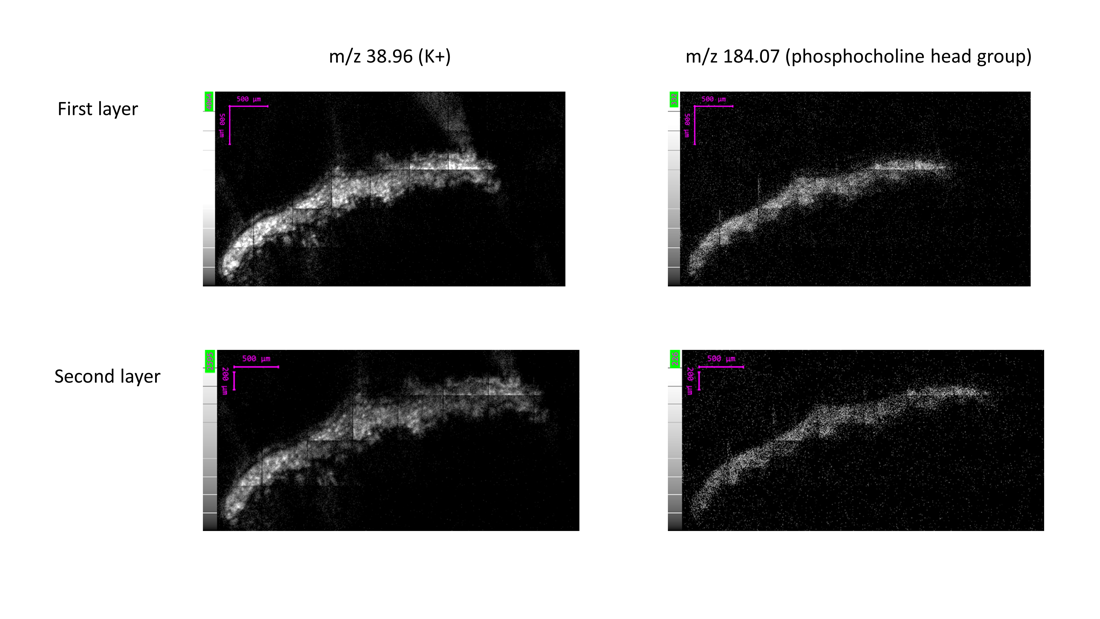


**Figure S8**. ToF-SIMS image of the same sections as in Figure 9. Here the same section has been repeatedly analysed. The first layer indicates the first imaging analysis of the cryosection, and second layer indicates a second imaging analysis of the same section. For representation, the ion images of *m/z* 38.96 and *m/z* 184.07 have been taken. It can be seen that the average ion intensity of the two ion signals decreases when going from layer 1 to 2. The average K^+^ intensity changes from 4664 to 2595, and the average phosphocholine signal intensity changes from 332 to 226.
